# Abdominal and Bowel Ultrasound Knowledge Among Young Gastroenterologists: Results of an Italian Survey

**DOI:** 10.3390/jcm14082693

**Published:** 2025-04-15

**Authors:** Fabio Cortellini, Anna Fichera, Alessia Dalila Guarino, Lucrezia Laterza, Luigina Vanessa Alemanni, Loris Lopetuso, Giovanni Marasco, Andrea Costantino

**Affiliations:** 1 Gastroenteroloy Unit, Azienda Unità Sanitaria Locale (AUSL) della Romagna Ospedale Infermi, 47921 Rimini, Italy; cortellinifabio@gmail.com; 2 Gastroenteroloy and Endoscopy Unit, Fondazione IRCCS Ca’ Granda Ospedale Maggiore Policlinico, 20122 Milan, Italy; ann.f@hotmail.it (A.F.); andrea.costantino@policlinico.mi.it (A.C.); 3 Gastroenterology Unit, Department of Clinical Medicine and Surgery, Università degli Studi di Napoli Federico II, 80146 Naples, Italy; alessiadalila.guarino@unina.it; 4 Centro Malattie Apparato Digerente (CEMAD) Digestive Disease Center, Fondazione Policlinico Universitario A. Gemelli IRCCS, Università Cattolica del Sacro Cuore, 00168 Rome, Italy; lucrezia.laterza@policlinicogemelli.it (L.L.); lopetusoloris@libero.it (L.L.); 5 Gastroenteroloy Unit, Azienda Unità Sanitaria Locale (AUSL) della Romagna, Ospedale Morgani-Pierantoni, 47121 Forlì, Italy; vanessaalemanni1@gmail.com; 6 Department of Medical and Surgical Sciences, University of Bologna, 40138 Bologna, Italy

**Keywords:** abdominal ultrasound, gastroenterology, education

## Abstract

**Background**: The diagnostic accuracy of abdominal ultrasound (US) is operator-dependent and, therefore, influenced by inadequate training and lack of continuous medical education. To fill this gap, the European Federation of Societies for Ultrasound in Medicine and Biology (EFSUMB) has developed guidelines to identify minimum training requirements for US. The aim of our survey was to assess the self-reported overall US education level among young Italian gastroenterologists. **Methods**: The Italian Association of Young Gastroenterologists and Endoscopists (Associazione Giovani Gastroenterologi ed Endoscopisti Italiani, AGGEI) developed a web-based survey with a multiple-choice test with images, based on the EFSUMB recommendations. The survey was distributed via e-mail to AGGEI members. **Results**: The questionnaire was filled out by 110 participants from all over Italy. Most of the respondents worked in academic hospitals and were gastroenterology residents or PhD students. More than half (58.9%) learned US during their gastroenterology training and 8.2% attended specific courses. During their training participants performed a median number of 320 abdominal USs and 240 bowel USs. Participants receiving a longer training period ranked significantly better in the knowledge questionnaire. **Conclusions**: Young Italian gastroenterologists show heterogeneous training in residencies across the country. In the future learning and hands-on training courses endorsed by academies are needed to fill this knowledge and skill gap.

## 1. Introduction

Ultrasound (US) is now part of routine clinical and outpatient practice. Indeed, its large availability, low costs, and the absence of radiation risks have made US a common tool in clinical practice as a first-line diagnostic technique [1,2]. In the gastroenterological setting, the clinical value of US yielded high diagnostic accuracy for the diagnosis of several diseases [3,4,5,6,7]. In addition, bowel US plays an important role in the diagnosis of inflammatory bowel diseases (IBDs), their complications, and management, including early identification of post-operative recurrence (POR) in Crohn’s disease (CD), as underlined by the European Crohn’s and Colitis Organization–European Society of Gastrointestinal and Abdominal Radiology (ECCO–ESGAR) guidelines [8,9,10,11,12].

Advanced techniques such as contrast-enhanced ultrasound (CEUS) and SICUS (small-intestine contrast ultrasonography) have ameliorated the US approach, allowing functional and vascular information to be obtained in real time. In addition to these applications, operative abdominal US plays an increasingly important role in minimally invasive interventional procedures, such as US-guided biopsies [13,14,15,16], drainage of abdominal collections, and thermal ablations of liver lesions [17,18,19].

Nevertheless, US, like many other diagnostic procedures, is operator-dependent, and its variable accuracy may lead to diagnostic mistakes in the absence of adequate training and continuous medical education [20].

The European Federation of Societies for Ultrasound in Medicine and Biology (EFSUMB) has developed guidelines to identify professional standards and minimum training requirements for US examination. Three levels of expertise have been determined according to the knowledge of anatomy, disease assessment by US examination, and the number of exams performed (300 USs per year for level 1) [20,21]. Similarly, the Italian Society of Medical Ultrasound (Società Italiana di Ultrasonologia in Medicina e Biologia, SIUMB) has published a position paper on the standardization of US technique and interpretation of abdominal US to overcome the growing discrepancy among operators [22].

As specified in the above-mentioned official statements, US knowledge consists of theoretical education covering physics principles of US, image recording, reporting, artifacts and the relevance of other imaging modalities to US, interpretational capacities of the standard US anatomy, and the principal pathological conditions, technical skills to obtain clear and diagnostic scans [20,21,22]. To improve the diagnostic accuracy of abdominal US, young doctors must receive adequate training, combining theory and clinical practice. Only through education, the use of US can be ensured, thereby improving the quality of healthcare. The adoption of structured training programs and the use of advanced simulators can facilitate learning. In addition, participation in workshops and refresher courses allows young doctors to hone their skills and stay up to date on technological innovations in the US field [20,21,22,23,24]. However, US training relies mainly on heterogeneous periods characterized by non-standardized time and quality of teaching during medical training and/or on individuals attending optional focused courses. At present, no data are available regarding the current level of training, skills, or competencies of trainees. This gap in information makes it challenging to identify areas for improvement and develop targeted educational strategies to enhance their professional expertise [24]. Therefore, we aimed to identify the current education level among young Italian gastroenterologists in US of the digestive system.

## 2. Materials and Methods

### 2.1. Study Design

The Steering Committee of Ultrasound section of the Italian Association of Young Gastroenterologists and Endoscopists (AGGEI) developed and internally validated a web-based survey during 4 videoconferences.

The committee tested a first draft of the questionnaire with a small sample of 10 participants (60% physicians) for initial validation. Considering that 40% of respondents were medical students or other healthcare professionals (nurses/nutritionists), two questions which received 100% correct responses were changed as they were considered too easy for the average 1st–4th-year gastroenterology residents. The final version of the US questionnaire was approved by a group of four experienced abdominal sonographers according to EFSUMB guidelines (19).

The survey was finally approved by all the members of the AGGEI Steering Committee.

### 2.2. Survey Development and Distribution

The questionnaire consisted of 36 multiple-choice questions, including a short survey and a multiple-choice test on US images. The survey assessed demographic characteristics and US education of the participants, as questions were based on the EFSUMB recommendations on the minimum training requirements for an US examination (see Appendices 1, 2, and 5). In the multiple-choice test, the following fields were explored: (a) US physics and instrumentation, (b) US diagnostic appropriateness, (c) image recording and reporting. Moreover, principles of (d) upper abdominal US anatomy (e) and US pathological images of the liver, gallbladder, bile ducts, pancreas, portal vessels, and spleen were also examined. All the scans were collected by two task force members during their clinical practice and saved anonymously, with previous written informed consent obtained from the patients. The multiple-choice options and the correct answers were defined by a consensus between all the members of the AGGEI Steering Committee. The members of the committee are all consultant gastroenterologists specialized in bowel ultrasound (BUS) and upper abdominal ultrasound (UAUS) who perform US daily. The complete version of the questionnaire is available in Supplementary Materials File S1.

The electronic version of the questionnaire was distributed via e-mail to all the members of AGGEI during the annual meeting that was held in November 2021 and was accessible online during the following 3 months. All respondents answered voluntarily, without any secondary rewards, and gave their consent to collection and analysis of the data for scientific purposes.

### 2.3. Statistical Analysis

The data are presented as counts and percentages for the categorical variables and mean and standard deviation (SD) for the continuous variables. Respondents were subdivided into two groups, namely low- and high-score respondents, in accordance with the median value of correct answers. Among the two groups, the categorical variables were compared using the Chi-squared or Fisher’s exact tests. For multiple categorical variables, the Chi-squared test of independence was used. Uni- and multivariate logistic regression models were performed to assess factors associated with high scores on the US images quiz. The results were accounted with an odds ratio (OR) with 95% confidence intervals (95% CI). An OR with an entire 95% CI less than 1 indicated that the covariate reduced the risk of the event; on the other hand, when the OR with an entire 95% CI was higher than 1, the covariate increased the risk of the event. An OR with a 95% CI across to 1 implicated that the covariate did not significantly influence the risk. The probability values were two-sided; a probability value of less than 0.05 was considered statistically significant. We carried out the statistical analysis with STATA 17.0 (College Station, TX, USA: StataCorp LP).

## 3. Results

### 3.1. Demographics

Among all the young (<40 years old) Italian gastroenterologists invited, 110 completed the survey (110/300, 36.7%) and were included in the final analysis. The characteristics of the respondents included in the study are summarized in Table 1.

The majority of participants were males (60/110, 54.5%). More than half of the participants (54.5%) were aged <30 years. Thirty-nine percent (39%) of participants in the survey were from the south and the islands. The remaining respondents were from: the north-east (32.7%), north-west (20%), and center (8.2%). Three-quarters worked in academic hospitals (74.5%) and most were gastroenterology trainees or PhD students (71.8%).

### 3.2. US Training Characteristics

Fifty-nine percent (59%) of the participants did not feel confident in completing exams independently. During their training, participants performed overall 320 (median; interval quartile range, IQR 0–1280) upper abdominal US and 240 (median; IQR 0–640) intestinal US examinations. More than half of the participants (55%) performed more than 300 exams per year (EFSUMB level 1). The majority of participants reported to have completed US training during the medical specialization (65, 58.9%), 12 (21.4%) in their workplace by colleagues, and nine (16.1%) attended specific US courses. Finally, nine out of 10 respondents had an ultrasound service in their unit and 74.5% believed that US is an essential skill for a young gastroenterologist.

### 3.3. US Images Tests

The results of the US images questionnaire are reported in Table 2. In total, the participants correctly answered 76% of the multiple-choice questions.

Optimal scores were found in physics and US anatomy, with 83.6% and 78.2% correct answers, respectively. In the section regarding common gastroenterological US pathological images, respondents properly interpreted 82.7% of liver US scans. Good results were obtained in the biliary system section (70.9% exact answers), while lower scores were observed in the pancreas and bowel sections (37.3% and 45.4%, respectively). Respondents were divided in two groups according to their median results, where at least 14/22 questions were necessary to be assigned to the high-score group. Sixty-five participants (59%) reached 14 or more correct answers in the multiple-choice quiz and were placed in the high-score group, while the remaining 45 (41%) were in the low-score group. Gastroenterologists > 30 years old were mainly in the high-score group (72%), while among those younger (<30 years old) 52% achieved ≥14 right answers. The majority of non-academics (75%) and consultants (70%) were in the high-score group. (Table 3). We conducted univariate analyses to assess predictive factors for obtaining high scores, finding that younger age (OR 2.796, *p* = 0.013) and working in non-academic hospitals (OR 2.591, *p* = 0.052) were associated with higher scores. However, at multivariate analysis only younger-age participants resisted (OR 2.345, *p* = 0.052).

## 4. Discussion

In the present study we evaluated the current clinical practice and knowledge of abdominal US among a national cohort of young gastroenterologists from different Italian regions. As previously discussed, the US training period is not standardized during residency [22] and this leads to discrepancy in the level of diagnostic accuracy among gastroenterologists. Even if specific requirements are needed according to European and national guidelines [19,21,22], they are not systematically checked, and US skills rely on self-evaluation and confidence.

Other European countries have available national recommendations on US training for residents of medical and surgical specialties. For instance, the UK Royal College of Radiologists precisely claimed how the residents should be trained and the competencies they should acquire to be a radiologist competent in US. However, US is not performed only by radiologists anymore, as for clinicians, including gastroenterologists, it has become the natural integration of the physical examination in a digital era. In this paper, we investigated the current level of competence in Italian young gastroenterologists according to the minimum number of supervised USs for a resident (250 for level 1) and the advised standard for training [23].

Our survey reveals that only 58.9% of participants learned US during their training, while a non-negligible group (21.4%) of gastroenterologists acquired this skill after their residency and another small group attended specific fee-based courses on an individual basis. US could improve the learning of anatomy facilitating in vivo representation of organs in a such preliminary phase of the study of medicine. At the same time, an early use of US will facilitate its use in the daily clinical practice of future physicians [24].

As predictable, our study shows that participants older than 30 years (45.5%) had a better competence in US, as they belong to the high-score group (*p* < 0.021), but interestingly not all of them were consultants, suggesting that clinical experience and training are the cornerstone in US knowledge. Surprisingly, a statistical difference was not observed in terms of high scores gained between gastroenterology residents and consultants.

In recent years, the interest in US has increased and the potential advantages of US teaching in preclinical and clinical settings led to the debate as to when is the right time to start US training. Considering that US could improve understanding of anatomy, physiology, and pathology, it has been proposed that US should be taught to undergraduate students. [25] Several studies have shown that US courses can improve the ability of medical students to recognize anatomical structures and simple pathological findings [26,27,28,29], with no difference whether they are delivered by peers or skilled trainers [26,27,28,29,30], and this evidence could facilitate the implementation of US training, even only peer-based, to contain costs and support a widespread diffusion of US knowledge. The possibility of learning US was highly appreciated by medical students who were asked to answer a survey during their first year after a theoretic session on principles and basics in US [31]. They answered that US training would lead to better knowledge of internal medicine and diagnostic methods and their confidence in clinical decision-making. However, this new possibility of learning opens many questions about how to realize this training with adequate standards and, notwithstanding the enthusiasm of students and the recognized utility of US training in the position papers, the US training is still far from being systematically part of medical teaching worldwide; moreover, as our survey demonstrated, even in a country with a long history of use of US, 21.4% of gastroenterologists should wait after residency to learn about US.

A similar scenario has been described in another country with a high specialization in US. In 2021, a survey among university chief gastroenterologists in Germany revealed that there was still high variability in the offered training among departments in terms of training duration, minimum number of requested exams, and amount of supervision, despite an increasing availability of interdisciplinary ultrasound centers with a capillary presence in national society in universities (96% of chief gastroenterologists were members of the German Society of Ultrasound in Medicine) [32]. 

Another interesting point outlined by our study is that participants from academic hospitals gained lower scores; this result may be not associated with a low level of expertise among university hospitals; instead, it may be influenced by the presence of young gastroenterology residents, probably less experienced than fully formed consultants from non-academic hospitals.

Of note, participants who affirmed to feel confident in performing abdominal ultrasound were independently more likely not to be in the high-score group. This association proved to be statistically significant both in univariate (*p* < 0.013) and multivariate analysis (*p* < 0.019) and should be interpreted as a sign that the self-perception of awareness does not necessarily reflect a real appropriate experience in the field of abdominal US. The presumption of being competent may have played a role in a superficial answering to the questionnaire, with self-confident participants paying less attention to the questions. Based on these findings, the possibility of introducing an US education in the early phase of gastroenterology residency will give the chance to trainees to have enough time during their curriculum to achieve minimum goals of quality in US. Awaiting more diffuse learning during medical school, to fill the knowledge bias of gastroenterology trainees without any previous specific US learning, teaching should first cover theoretical education on the physics principles and anatomy. Then, it should include access to an archive of multiple US videos and images to give a comprehensive US cultural background to the second hands-on part. Distance teaching through telemedicine methodologies showed similar quality compared to traditional methods and it could be helpful to ensure high-quality training also for trainees in peripheral centers [33]. The hands-on approach is typically a critical point in US apprenticeship. Probably in the future, the use of artificial models could overcome this issue, supporting the creation of US schools and increasing the hours of hands-on learning, a recognized clinical point in US training [34]. As a complementary alternative, gastroenterology trainees could be assigned in small groups to expert sonographers in the academic setting and likewise complete their training with other hours of peer-to-peer learning with senior trainees who have already completed their internship, but that could still benefit from a teach-the-teacher method. These recommendations will improve the quality of apprenticeship and will reduce the risk for future patients to be evaluated by gastroenterologists without adequate training.

It should be considered that in the future, it is likely that pocket-size US devices will further increase the use of US by gastroenterologists to answer simple clinical questions [35,36,37,38,39]. Of course, these points of care in abdominal and bowel US will be helpful for clinical decisions only if gastroenterologists are able to interpret images correctly.

For instance, if a physician producing inadequate images is not able to detect small quantities of fluid in the emergency room, this results in a change in the management that could put at risk patient outcome and care [40].

Our study has some limitations. For instance, one bias could be that being a dynamic examination, using an image-based questionnaire to establish US expertise may not accurately evaluate the ability to actually perform US.

Another limitation is the limited number of participants and the inclusion of gastroenterology residents with a short US training period. It could be related to a possible selection bias since members of AGGEI and participants in its national meeting are mostly young doctors during their residency or first-year post-residency work. However, such selection allowed us to collect a real-life-based experience and is therefore valuable for the data it provides on the role of US training. For example, it enables us to better characterize the areas in which trainee doctors first develop autonomy and awareness, and those in which they struggle most; such information could help us structure better US training programs in the future.

Most of all, our study highlights the role of experience in US performance. However, we are aware that a larger sample size would allow us to affirm this data with greater strength and statistical significance. Considering that the survey was web-based, time-consuming, and without compensation, we still consider our response rate as a source of precious information. More similar studies are needed to confirm our data and help US teaching programs improve.

Therefore, we still support the utility of our study, which reports an assessment of US knowledge among a national cohort of young gastroenterologists and residents.

## 5. Conclusions

This survey shows that US knowledge is considered as a necessary skill by ¾ of young gastroenterologists, but US training remains heterogeneous, suggesting the need for moving forward. Academies and the institution should integrate the gastroenterology formation plan with a basic US teaching and practice during residency to ensure the minimum standard of quality in US among future gastroenterologists.

## Figures and Tables

**Table 1:** Demographics and working characteristics of participants in the survey.

	*n* = 110	(%)
Age		
<30	60	(54.5)
<40	50	(45.5)
Males	60	(54.5)
Region		
Center	9	(8.2)
North-east	36	(32.7)
North-west	22	(20)
South and islands	43	(39.1)
Workplace		
Academic	82	(74.5)
Non-academic	28	(25.5)
Clinical role Gastroenterology resident Consultant	79 31	(71.8) (28.2)
Respondents who feel confident in performing abdominal or bowel US independently	45	(40.9)
US training		
Training during residency	35	(58.9)
Workplace	12	(21.4)
Specific US courses	9	(16.1)
US service in the gastroenterology unit	99	(90)

**Table 2:** Multiple-choice test on ultrasound knowledge results.

*n* tot = 110	Answers Collected (*n*)	Correct Answers	
		(*n*)	(%)
Background theoretical knowledge	97	92	83.6
Technical skills	96	63	57.3
Recording and reporting	84	58	52.7
Interpretational skills: US anatomy	96	86	78.2
Liver	95	91	82.7
Biliary system	94	78	70.9
Pancreas	85	41	37.3
Portal vein and spleen	89	65	59.1
Bowel and other	85	50	45.4

**Table 3:** Differences between the groups of high and low scores.

*n* = 110	Low Scores *n* = 45 (41%)		High Scores *n* = 65 (59%)		OR (95%CI) Univariate Analysis	Univariate Analysis (*p*)	OR (95%CI) Multivariate Analysis	Multivariate Analysis (*p*)
**Age**								
<30	31	(48%)	29	(52%)	2.796 (1.237–6.319)	0.013	2.345 (0.994–5.532)	0.052
<40	14	(28%)	36	(72%)	2.138 (0.184–24.856)	0.544		
**Gender**								
Females	18	(36%)	32	(64%)	-			
Males	27	(45%)	33	(55%)	1.5 (0.693–3.246)	0.303		
**Area of Italy**								
Center	2	(22%)	7	(78%)	-			
North-east	13	(36%)	23	(64%)	0.506 (0.091–2.801)	0.435		
North-west	27	(32%)	15	(68%)	0.612 (0.100–3.739)	0.595		
South and islands	23	(53%)	20	(47%)	0.248 (0.046–1.336)	0.105		
**Workplace**								
Academic	38	(46%)	44	(54%)	-			
Non-academic	7	(25%)	21	(75%)	2.591 (0.993–6.761)	0.052		
**Clinical Role**								
Resident	36	(46%)	43	(54%)	-			
Consultant	9	(30%)	22	(70%)	2.047 (0.838–4.999)	0.116		

## Data Availability

The datasets generated and/or analyzed during the current study are not publicly available but are available from the corresponding author upon reasonable request.

## Supplementary Materials

The following supporting information can be downloaded at https://www.mdpi.com/article/10.3390/jcm14082693/s1, File S1: Web-based survey with a multiple-choice test with images.

## Abbreviations

The following abbreviations are used in this manuscript:

US	Ultrasound
EFSUMB	European Federation of Societies for Ultrasound in Medicine and Biology
AGGEI	The Italian Association of Young Gastroenterologists and Endoscopists
IBD	Inflammatory bowel diseases
ECCO	European Crohn’s and Colitis Organization
ESGAR	European Society of Gastrointestinal and Abdominal Radiology
SIUMB	The Italian Society of Medical Ultrasound
BUS	Bowel ultrasound
UAUS	Upper abdominal ultrasound
SD	Standard deviation
OR	Odds ratio
CI	Confidence interval
IQR	Interval quartile range
