# Supplementary material for: Abdominal and Bowel Ultrasound Knowledge Among Young Gastroenterologists: Results of an Italian Survey
Source: J Clin Med. 2025 Apr 15;14(8):2693. doi: 10.3390/jcm14082693 (PMC12027906; doi:10.3390/jcm14082693)
Supplement: Supplementary file 1 [file jcm-14-02693-s001.zip › jcm-3448154-supplementary.pdf]

**Complete Survey Questions**

# Demographics

0. AGGEI/UEG member? If yes, did you participate in the ultrasound workshop during the campus?

1. Please select your gender:
   - Female
   - Male
2. Please select your age:

   - 1 to 40

3. Workplace
   - North-West (Valle d’Aosta, Piemonte, Lombardia, Liguria)
   - North-East (Friuli-Venezia Giulia, Veneto, Emilia Romagna, Provincia di Trento/ Bolzano)
   - Center (Toscana, Marche, Umbria, Lazio)
   - South and Islands (Abruzzo, Molise, Campania, Puglia, Basilicata, Calabria, Sicilia, Sardegna)

4. Where is your practice setting?
   - University hospital
   - Non-University hospital
   - Private setting
   - Other

5. What is your specialty?
   - Gastroenterology
   - Internal Medicine

6. What is your role?
   - Fellow in training
   - Physician
   - PhD

7. Do you feel confident in performing abdominal ultrasound exams independently?
   - No
   - Yes
   -
8. If yes, how did you learn?
   - Fellowship
   - Focused courses
   - Workplace

9. How long did it take? (months)
   -

10. How many upper abdomen ultrasound exams did you perform per week during your ultrasound practice period?
    - 20
    - 40

- 80
- 100

11. Is there in your workplace an ultrasound facility?
- Yes
- No, we refer exams to other units

12. Do you believe that it is essential for a young gastroenterologist to have the ability to do ultrasounds independently/on their own?
- absolutely essential
- useful but not essential
- not useful at all

13. If you have ever studied or practiced ultrasounds, continue to the next section, if not, this survey ends here.

# Ultrasound knowledge

**Physics and instrumentation**

1. Which kind of probe do you use for a normal upper abdomen ultrasound examination?
   - Convex probe 3,5-5,5 MHz
   - Linear probe 7,5-13 MHz
   - Sectorial probe 2,5-3,5 MHz

2. How can the ultrasound waves reach deeper tissues?
   - Low frequencies
   - High frequencies

**Ultrasound techniques (Indications, relevance to other imaging modalities, the influence of ultrasound results, and need for other imaging modalities)**

3. How frequently should undergo an upper abdomen ultrasound examination a patient with compensated chronic advanced liver disease?

   - Every 6 months
   - Every 3 months
   - Every 12 months

4. In the case of intrahepatic biliary dilatation found during ultrasound examination, what is the gold standard diagnostic exam of choice to discover the etiology?
   - Cholangio-MRN/EUS
   - Contrast-Enhanced CT
   - MRN with liver-specific contrast

5. In which of the following situations follow-up every 6 months with upper abdominal ultrasound should be done?
   - Cholecystic polyp $6 \geq x \leq 9$ mm, no risk factors
   - Cholecystic polyp $\leq 5$ mm, no risk factors
   - Cholecystic polyp $\geq 10$ mm

6. If you find this focal liver lesion in a patient without a history of malignant or chronic liver disease, which of the following is the correct management? (IMMAGINE ANGIOMA)
    - Benign focal liver lesion, no further evaluations
    - Focal liver lesion, CEUS is indicated
    - Focal liver lesion, B-mode follow-up every 6 months

**Administration**

7. Do you write a report after every exam?
    - No
    - Yes, only in routine examinations
    - Yes, also in bed-side point-of-care ultrasound

8. Do you attach images of standard scans? (printed or digitally stored) in a negative exam?
    - Yes
    - No

9. If so, which standard scans do you always include?
    - Hepatic vein plan, portal vein bifurcation plan, left liver lobe, right liver lobe with right kidney, liver hilum, gallbladder, pancreas, spleen, left kidney
    - Right liver lobe, left liver lobe, caudate lobe, gallbladder, pancreas, spleen, left kidney right kidney, splenic vein, portal vein, mesenteric vein
    - I do not include always the same

**Sectional and ultrasonic anatomy**

10. Select the organ scanned in the image below (IMMAGINE/CLIP VIDEO FEGATO CON SOVRAEPATICHE/BIFORCAZIONE PORTALE)
    - Liver
    - Spleen
    - Kidney
    - Pancreas

11. Select the organ scanned in the image below (IMMAGINE/CLIP VIDEO RENE)
    - Kidney
    - Pancreas
    - Small bowel
    - Liver
    - Adrenal gland

12. Choose the normal upper dimension limits of a spleen in an adult.
    - Bipolar diameter 12 cm, section area 45 cm2
    - Bipolar diameter 8 cm, section area 35 cm2
    - Bipolar diameter 13 cm, section area 60 cm2
    - Bipolar diameter 12 cm, section area 70 cm2
    - Bipolar diameter 7 cm, section area 30 cm2

**Pathology in relation to ultrasounds**

***Liver***

13. Which focal lesion stays between the calipers? (IMMAGINE CISTI EPATICA)
    - Simple hepatic cyst
    - Simple splenic cyst
    - Hepatic Hemangioma
    - Hepatic nodule
    - Gallbladder

14. Which pathological picture is reported in the following images? (IMMAGINI STEATOSI EPATICA)

- Hepatic steatosis
- Hepatic cirrhosis
- Adenomiomatosis
- Hepatic cyst
- Hepatic Hemangioma

***Biliary system***

15. Describe the following ultrasound scan: (CLIP VIDEO CALCOLI COLECISTI)

- Gallbladder lithiasis
- Choledochal lithiasis
- Acute cholecystitis
- Chronic cholecystitis
- Biliary sludge

16. Which of the following is not a suspected sign for acute cholecystitis?
    - "Comet-tail" reverberation artifacts
    - Gallbladder wall thickening > 6 mm
    - Pericholecystic inflammation or fluid
    - Antero-Posterior diameter of gallbladder > 4 cm
    - Hyperemia of the gallbladder wall at power-Doppler

***Portal venous system and spleen***

17. Which of the following could be a pathological finding?
    - Portal vein diameter of 9 mm
    - A-P diameter of Aorta of 20 mm
    - Splenic vein diameter of 11 mm
    - Common bile duct diameter of 5 mm
    - Wirsung diameter of 2 mm
    - (Mean portal flow velocity of 35 cm/sec at Doppler assessment)

18. Describe the following ultrasound scan: (IMMAGINE TROMBOSI PORTALE)

- Portal thrombosis
- Chronic pancreatitis
- Periportal venous collaterals
- Portal ectasia

19. Describe the following ultrasound scan: (IMMAGINE VERSAMENTO PERIEPATICO)

    - Hepatic ascites

- Splenic ascites
- Hepatic steatosis
- Splenic vein thrombosis
- Budd-Chiari syndrome

***Pancreas***

20. Which pathological picture is reported in the following image? (IMMAGINE PANCREATITE CRONICA)
    - Chronic pancreatitis
    - Acute pancreatitis
    - Main pancreatic duct obstruction
    - Acute cholecystitis
    - Intestinal obstruction

***Bowel and other***

21. Describe the following ultrasound scan: (IMMAGINE LINFONODI)
    - inflammatory lymph nodes
    - malignant lymph nodes
    - atrophic kidney
    - adrenal gland

22. Which of these is not a common ultrasound artifact?
    - Acoustic posterior enhancement
    - Mirror images
    - Reverberation
    - Double Aorta appearance
    - All the answers refer to ultrasound artifacts
